# Robustness of oxygen metrics to sampling frequency in a stratified lake

**DOI:** 10.1007/s10661-026-16081-8

**Published:** 2026-10-21

**Authors:** Eric Weniger, Ruben Sommaruga

**Affiliations:** Department of Ecology, Universität Innsbruck, Technikerstr. 25, 6020 Innsbruck, Austria

**Keywords:** Dissolved oxygen, Lake monitoring, Temporal interpolation, Oxygen depletion rate, High-frequency sensors

## Abstract

Long-term lake monitoring often relies on monthly dissolved oxygen profiles, but the robustness of derived oxygen metrics to this temporal resolution is uncertain. We compared three years (2022–2024) of hourly sensor observations (high-frequency (HF)) with approximately monthly discrete profiles (low-frequency (LF)) from a small, dimictic subalpine lake. We evaluated volume-weighted oxygen concentrations, hypolimnetic oxygen depletion rates (ODR) and depth-specific counts of days with oxygen < 2 mg L⁻^1^. To isolate temporal sampling effects on ODR, we subsampled the HF daily record at 7-, 14-, 30- and 60-day intervals across all starting-day offsets within fixed annual fitting periods. LF profiles interpolated to daily resolution reproduced broad seasonal oxygen trajectories, but concentration differences persisted on matched sampling dates, particularly at 9 and 12 m. The mean within-year range width of signed ODR deviations from the full-HF reference was 8.0 percentage points at 7-day sampling, 20.5 at 14 days, 47.5 at 30 days and 48.6 at 60 days. Depth-specific low-oxygen-day counts were strongly correlated between datasets (Pearson’s *r* = 0.933–0.977), but unequal observation coverage precluded unbiased estimates of annual duration. The results indicate that approximately monthly profiles can describe broad seasonal oxygen dynamics in this lake, whereas more frequent sampling reduces the sensitivity of ODR estimates to sampling timing.

**Supplementary Information:**

The online version contains supplementary material available at https://doi.org/10.1007/s10661-026-16081-8.

## Introduction

Dissolved oxygen (DO) is one of the most informative state variables in lakes because it links physical forcing, biological metabolism, redox chemistry, and habitat quality. Loss of oxygen restricts habitat for aerobic organisms, alters sediment–water exchange and favours the mobilization or transformation of redox-sensitive compounds (Lewis et al., 2024). Oxygen depletion is therefore central to assessments of eutrophication, restoration success and climate change effects (Adrian et al., 2009). Multilake analyses have documented widespread deoxygenation in temperate lakes, with deep water oxygen losses associated with stronger stratification and changes in water clarity (Jane et al., 2021). This is consistent with projections that warming will further lengthen stratification and extend hypolimnetic oxygen depletion (Woolway et al., 2021).

Detecting such changes depends on the temporal resolution of monitoring data. Long-term limnological programmes commonly rely on monthly or seasonal depth profiles because boat-based sampling is labour-intensive, weather-dependent and difficult to sustain at higher frequency. Such records remain essential for assessing long-term oxygen trends that started at times when data loggers were not available. Discrete profiles are typically interpolated across time and depth. This is reasonable when oxygen trajectories are smooth relative to the sampling interval, but uncertainty increases when concentrations change rapidly between observations. The monitoring question is therefore which oxygen metrics are robust to sparse sampling and which require higher temporal resolution (Patil et al., 2024, Rozemeijer et al., 2025).

Automated high-frequency sensors provide near-continuous observations of lake physics and chemistry and can resolve diel cycles, short-lived mixing events and rapid oxygen drawdown that discrete sampling may miss (Marcé et al., 2016; Meinson et al., 2016). They can also support management by detecting short-lived water quality events (Seifert-Dähnn et al., 2021). However, high-frequency monitoring requires instrumentation, maintenance, calibration and quality control (Marcé et al., 2016). Its value may therefore differ among monitoring objectives, making empirical assessment of resolution sensitivity important.

Stratified lakes provide a useful framework because the controls on oxygen dynamics vary with depth. In the epilimnion, oxygen reflects air–water gas exchange, photosynthesis and wind-driven mixing, while in the hypolimnion, it is largely controlled by respiration, decomposition and sediment oxygen demand during stratification (Matzinger et al., 2010; Wetzel, 2001). The metalimnion is characterized by steep density gradients that restrict vertical exchange, but can be perturbed by internal seiches, shear and intermittent entrainment. Biogeochemical activity in this zone can also generate local oxygen maxima and steep oxygen gradients (Giling et al., 2017; Weniger & Sommaruga, 2025).

Derived oxygen metrics can differ in their sensitivity to sampling frequency. Depth-specific counts of days below an oxygen threshold integrate conditions over time, whereas rate-based metrics such as hypolimnetic oxygen depletion rates depend on capturing the timing and slope of oxygen loss (Ladwig et al., 2021; Matzinger et al., 2010; Müller et al., 2012; Nürnberg, 1995).

Here, we compare at seasonal and interannual scales, daily oxygen time series derived from hourly logger measurements and approximately monthly depth profiles in a dimictic lake with pronounced hypolimnetic oxygen depletion. We assess how reduced temporal resolution affects (1) seasonal, layer-specific oxygen dynamics; (2) hypolimnetic oxygen depletion rates (ODR) and (3) depth-specific counts of days with oxygen < 2 mg L⁻^1^. We distinguish broad temporal agreement from sensitivity at shorter sampling intervals because sparse observations may reproduce a seasonal trajectory while missing short-lived changes that affect rate estimates. We hypothesised that (1) low-frequency profiles would reproduce broad seasonal changes in volume-weighted layer means, but show larger discrepancies at depths near strong gradients; (2) the vertical pattern of low-oxygen exposure would agree more strongly between HF and LF datasets than absolute duration and (3) ODR estimates would become more sensitive as the sampling interval increased. This design identifies which oxygen metrics can be recovered from historical low-frequency records and which require denser sampling.

## Methods

### Study site

Lake Piburg (Piburger See) is a subalpine lake in the Ötztal Alps of Tyrol, Austria (47°11′N; 10°53′E), at 913 m a.s.l. It has a surface area of ca. 0.13 km^2^ and a maximum depth of about 24.6 m and is surrounded predominantly by forested slopes. The lake undergoes pronounced seasonal thermal stratification, with a relatively stable hypolimnion during this period and complete or near-complete overturn during spring and autumn mixing (Niedrist et al., 2018). Because this study examines one small, dimictic, seasonally stratified lake with pronounced vertical oxygen gradients, the results should be interpreted as system-specific rather than representative of lakes generally.

Lake Piburg is a long-term socioecological research site (LTSER) with more than 50 years of monthly, depth-resolved limnological measurements, including water temperature and dissolved oxygen. This record provides a long low-frequency series against which more recent high-frequency observations can be evaluated. Previous work documented eutrophication, subsequent restoration, renewed re-eutrophication and increasing hypolimnetic oxygen depletion (Weniger & Sommaruga, 2025). Oxygen is therefore a useful indicator of the lake’s current ecological state. Depletion can proceed gradually during prolonged stratification, but may also change over shorter intervals, making Lake Piburg suitable for testing how sparse observations represent continuous oxygen dynamics.

### Data source

The low-frequency (LF) dataset comprised 36 discrete-depth profiles collected approximately monthly from January 2022 to December 2024 (Supplementary Table S1). Profiles were sampled at 3 m depth intervals from the surface (upper 45 cm) to 24 m using a modified 5-L Schindler–Patalas sampler with a 45-cm sampling height and an internal thermometer. Samples were immediately fixed with manganese sulfate and alkaline potassium iodide and transported to the laboratory, where dissolved oxygen was measured by Winkler titration.

Exact LF sampling times were unavailable in the archived dataset; thus, same-date HF–LF comparisons therefore used HF daytime means rather than coincident observations. The high-frequency (HF) dataset comprised in situ dissolved oxygen and water temperature measurements recorded hourly by HOBO U26 loggers (reported accuracy 0.2 mg L⁻^1^) deployed on a suspended line at 1, 3, 6, 9, 12, 15, 18, 21, 22 and 24 m depth. Logger availability varied among deployments; 22-m measurements were available through April 2024, but not during the final deployment (May–December 2024).

Handling the suspended line on 12 May 2022 coincided with an abrupt step at 11:00 CEST (09:00 UTC) in oxygen and temperature at 6, 9, 12 and 15 m depth. The line had been entangled from the start of the 2022 deployment, displacing the affected loggers upward. This entanglement was resolved on 12 May, after which the loggers were lowered. We aligned pre-inspection observations to the post-inspection reference using sensor- and variable-specific additive offsets (Fig. S1). For each affected sensor, the offset was the difference between the median of the 24 hourly observations beginning at 11:00 on 12 May and the median of the preceding 24 observations. Oxygen offsets at 6, 9, 12 and 15 m were − 1.160, − 1.895, − 3.700 and − 3.345 mg L⁻^1^, respectively, and corresponding temperature offsets were − 6.320, − 1.460, − 0.440 and − 0.160 °C. Offsets were applied only to that sensor’s 2022 observations before 11:00 on 12 May. Later observations, other depths and 2023–2024 deployments were unchanged. The correction assumes a constant additive displacement throughout the pre-inspection period and does not reconstruct unmeasured depths or changes in vertical gradients. Original values were retained alongside corrected values.

After correction, 12 oxygen observations at 12 m and 234 at 15 m became negative and were treated as missing and they were not replaced with zero. Their original and corrected values were retained in the quality control record. Corrected hourly oxygen profiles were linearly interpolated to a common 1-m depth grid without extrapolation, while temperature profiles were processed separately. The interpolated profiles were then averaged to daily values for HF analyses.

The LF profiles were also linearly interpolated to the common 1-m grid. Linear interpolation was used vertically because cubic splines can produce larger errors in heterogeneous water column profiles, particularly across steep concentration gradients (North & Livingstone, 2013). At each 1-m depth, LF observations were then interpolated in time to daily resolution using cubic splines. This approach was chosen to represent a smooth trajectory between monthly observations and is consistent with previous reconstructions of temporally sparse limnological profiles (North et al., 2014; Rempfer et al., 2009). No short-lived oxygen fluctuations between sampling dates are resolved by the reconstructed series.

For each water operational thermal layer (i.e., epi-, meta- and hypolimnion) and day, volume-weighted mean dissolved oxygen concentration was calculated as the sum of depth-specific oxygen concentrations weighted by the corresponding layer volume, divided by the total volume of the respective layer. These fixed operational zones were used for comparability across years and datasets and should not be interpreted as daily estimates of the physically shifting thermal layers. All analyses were conducted in R (version ≥ 4.3; R Core Team, 2024).

### Definition of thermal layers

Thermal layers were defined from daily temperature profiles and used to establish fixed depth intervals for subsequent oxygen analyses. HF temperatures were averaged by depth and day, while LF temperatures were taken from the temporally interpolated daily profiles. Both datasets were represented on the common 1-m depth grid.

For each daily profile, absolute temperature differences between adjacent 1-m intervals were calculated. Intervals with a temperature difference > 1 °C m⁻^1^ were classified as strong temperature gradients. Profiles were retained when the mean temperature at shallow depths (≤ 3 m) exceeded that at deep depths (≥ 21 m) and at least one interval exceeded the gradient threshold. Profiles meeting neither criterion were excluded from estimation of representative thermal boundaries. The > 1 °C m⁻^1^ threshold was used as an operational criterion for strong thermal gradients (Wetzel, 2001).

For each retained profile, the shallowest and deepest qualifying intervals were identified and represented by their deeper endpoints. The range between these endpoints was treated as the metalimnion. Representative upper and lower boundaries were calculated as median endpoint depths across qualifying profiles in 2022–2024, separately for HF and LF datasets; the 25th and 75th percentiles describe temporal variation (Table S2). In both datasets, median boundaries were 4 and 9 m. We therefore used fixed analytical intervals of 0–3 m (epilimnion), 4–9 m (metalimnion) and ≥ 10 m (hypolimnion). These are operational depth intervals derived from the observed thermal structure and were held constant across datasets and years, but they do not represent daily movement of the physical layers.

A generalized additive model (GAM) was used to describe broad temporal patterns in HF oxygen concentrations (Supplementary Methods). The model was used only for descriptive visualization and did not enter any HF–LF agreement metric or depletion rate calculation.

### Comparison of time series

For daily layer means, Pearson’s product–moment correlation coefficient (*r*), root mean square error (RMSE) and mean bias (HF − LF) were calculated from paired daily HF and LF volume-weighted oxygen concentrations, separately by thermal layer. Pearson’s *r* was used descriptively because temporal autocorrelation and LF interpolation mean that paired daily values are not independent observations.

To separate differences present on LF sampling dates from those introduced by temporal interpolation, original LF oxygen measurements were paired with HF observations on the same calendar date and nominal depth (3, 6, 9, 12, 15, 18, 21 and 24 m). No interpolation was applied to LF observations. HF oxygen was averaged from 08:00 to 17:00 local time (Europe/Vienna) because exact LF sampling times were unavailable. Pearson’s *r*, RMSE and mean bias (HF − LF) were used descriptively. Because these HF values are daytime means rather than coincident measurements, this comparison tests whether discrepancies persist at the sampling-date scale, but does not establish simultaneous measurement.

### Sensitivity of ODR to HF sampling interval

Annual ODR fitting windows were selected by visual inspection of the HF hypolimnetic oxygen time series. Each window represented sustained, approximately linear oxygen depletion from the onset of decline until near-anoxic conditions, reoxygenation, or a plateau was reached. ODR was estimated as the slope of an ordinary least-squares regression of daily, volume-weighted hypolimnetic oxygen concentration (depth ≥ 10 m) against elapsed time. ODR is expressed in mg L⁻^1^ d⁻^1^ and negative slopes indicate depletion. The initial windows were 16 April–15 July 2022, 12 February–9 June 2023, and 10 April–23 July 2024.

Only the portions of these windows with data available for both HF subsampling and the supplementary ODR comparison were analysed. The fixed fitting periods were 25 April to 15 July 2022, 12 February to 9 June 2023, and 11 April to 23 July 2024, with 82, 118 and 88 available HF daily observations, respectively. These periods were used for all subsampling schedules. The full-HF reference ODR was calculated from all available daily observations, and the same series was then subsampled at 7-, 14-, 30- and 60-day intervals.

For an interval of *k* days, the first sampling date was shifted successively from day 1 to day *k* of each fitting period, retaining every *k*th day thereafter. Only observations on scheduled dates within the fitting period were retained; missing HF observations were omitted without substitution, interpolation or extrapolation. ODR was estimated for each schedule by the same regression procedure, with at least two observations required. The starting-day offsets therefore represent the dependence of the estimated slope on sampling timing.

Signed percentage deviation from the full-HF reference was calculated as 100 × (ODRsubsample/ODRHF − 1). Because reference slopes are negative, positive deviations indicate faster depletion and negative deviations indicate slower depletion. For each interval, the minimum and maximum signed deviations were reported for each year. Within-year range width was calculated as maximum minus minimum deviation, and the three yearly widths were averaged with equal weight and expressed in percentage points. This analysis describes sensitivity to sampling timing within a known depletion period and retains gaps in the HF record.

### Supplementary ODR comparison

A second comparison used the actual LF sampling dates. This comparison combines differences in sampling frequency with differences in measurement method (in situ oxygen sensors versus Schindler–Patalas sampling and Winkler titration). Its detailed methods are presented in the supplementary information (Supplementary Methods).

### Depth-specific duration of low-oxygen conditions

Low-oxygen conditions were defined as dissolved oxygen < 2 mg L⁻^1^. For each dataset, daily depth-specific oxygen concentrations were classified as below or above this threshold, and the number of low-oxygen days was calculated for each depth and year (2022–2024). Because HF daily values exist only for days with sensor observations, the HF dataset contains temporal gaps, whereas LF values are interpolated within the available profile record. Calendar year coverage therefore differs between datasets. To compare the vertical pattern of exposure, depth-wise counts were paired by year and depth. Pearson’s *r*, RMSE, and mean bias (LF − HF) were calculated separately for each year and used descriptively. Unequal coverage means that these counts should be interpreted as comparisons of the vertical exposure pattern, not as unbiased estimates of annual duration. Further, depth-wise correlations were interpreted descriptively, without significance tests, because adjacent depths within a reconstructed profile are not independent replicates.

## Results

### The HF time series

HF oxygen concentrations showed clear seasonal dynamics (Fig. S2). Epilimnetic and metalimnetic concentrations generally increased during spring and summer and declined towards winter, whereas hypolimnetic concentrations declined during depletion periods with intermittent reoxygenation. The smooth terms for all three depth zones were significant (*p* < 0.001).

### Comparison of HF and LF time series

**Fig. 1:**
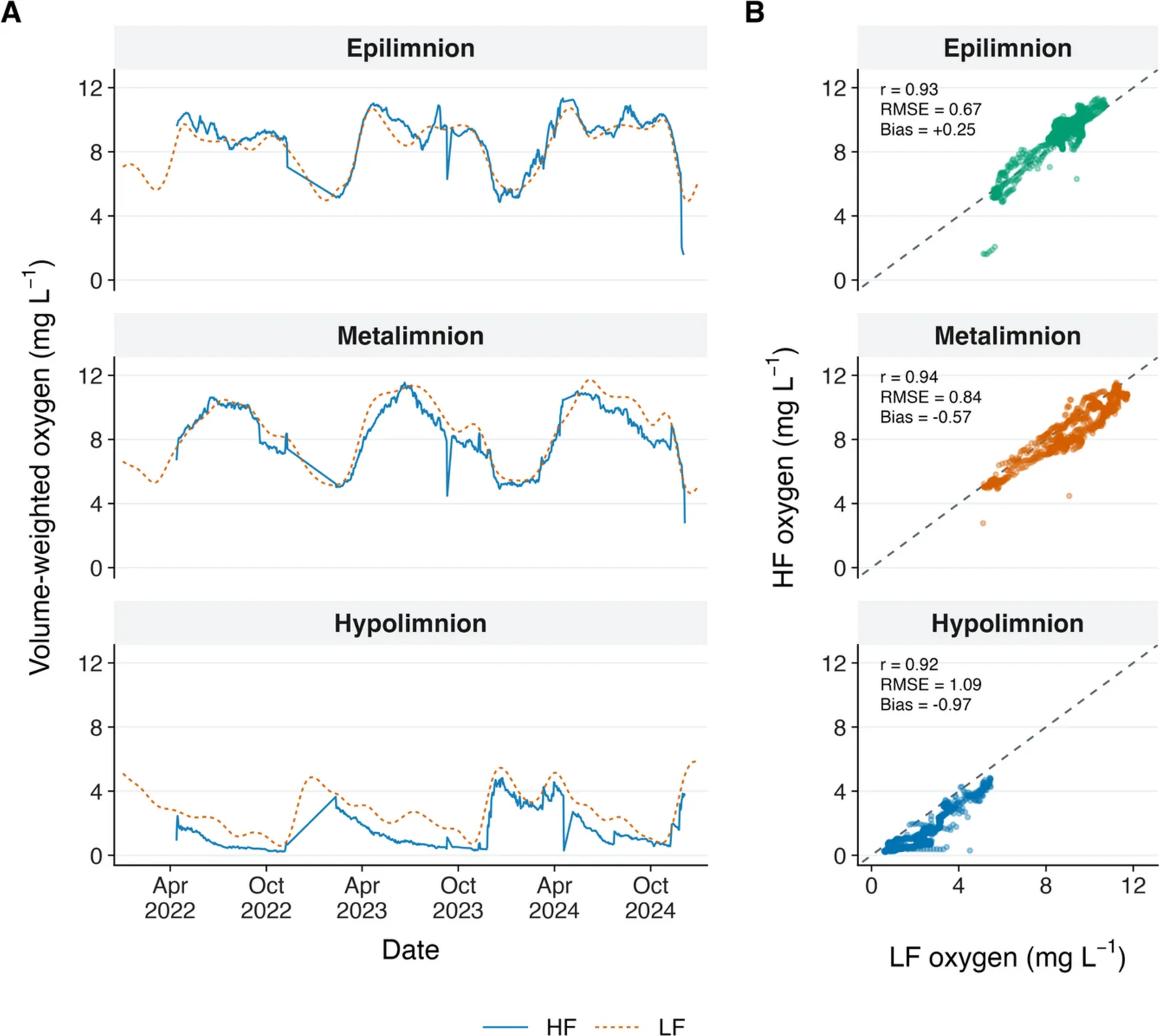
**A** Daily volume-weighted oxygen concentrations from HF observations and the monthly-interpolated LF series in the depth zones 0–3, 4–9 and ≥ 10 m. Solid lines denote HF daily means; dashed lines denote the monthly-interpolated LF series. **B** Paired daily LF and HF concentrations by lake layer; the dashed line indicates 1:1 agreement. Here, r denotes Pearson’s correlation coefficient. Bias is HF–LF; RMSE and bias are in mg L⁻^1^

The approximately monthly LF series reproduced the broad seasonal variation of the HF record in all three operational layers (Fig. 1), but systematic concentration offsets persisted.

Daily LF–HF Pearson correlations were 0.928, 0.945 and 0.916 for the epilimnion, metalimnion and hypolimnion, respectively. RMSE values were 0.67, 0.84 and 1.09 mg L⁻^1^, and mean biases (HF–LF) were + 0.25, − 0.57 and − 0.97 mg L⁻^1^. Correlation was highest in the metalimnion and lowest in the hypolimnion, where the negative bias was also largest.

### Sensitivity of ODR to HF sampling interval

**Fig. 2:**
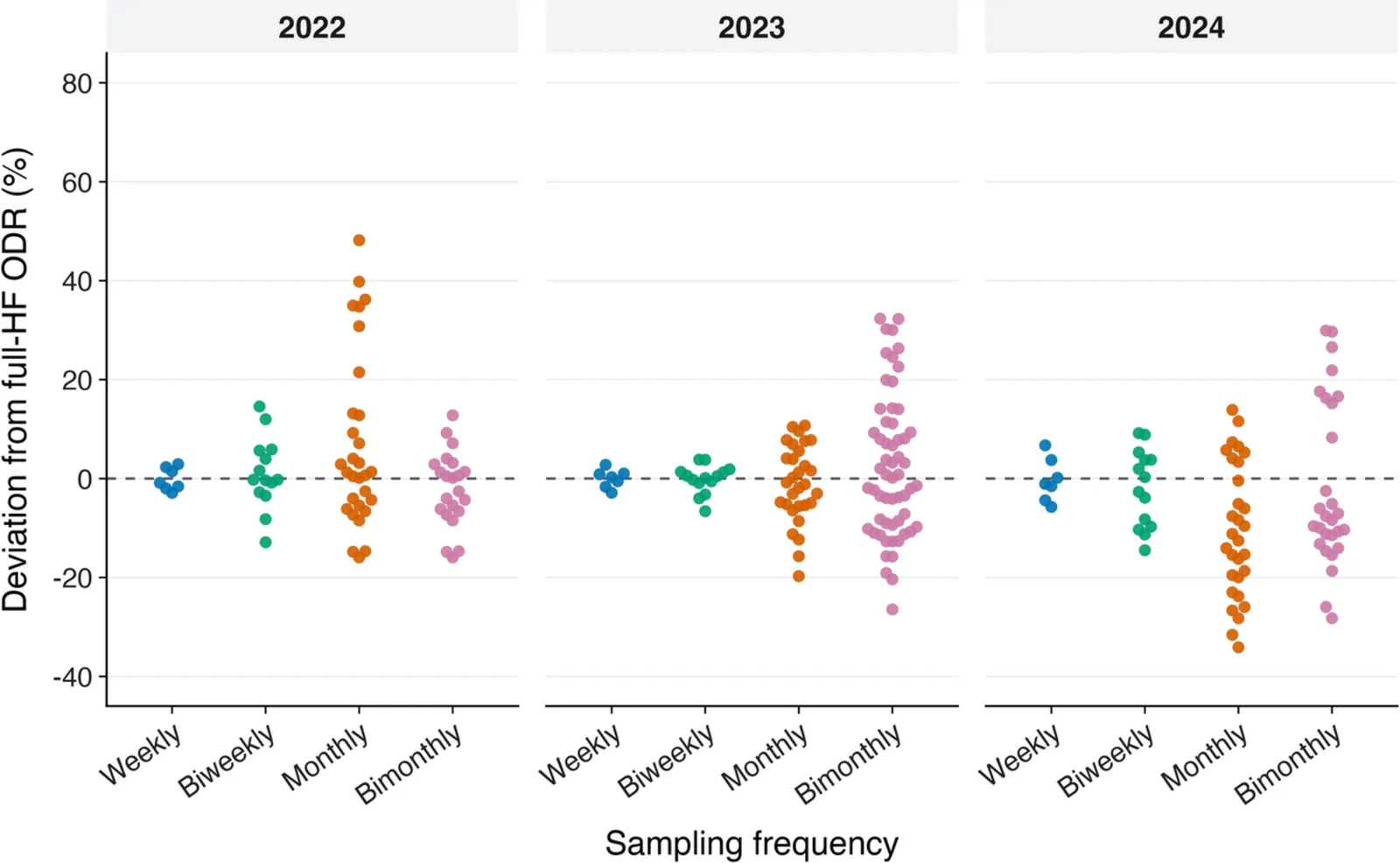
Sensitivity of hypolimnetic oxygen depletion rates (ODR) to sampling interval. Each circle shows the signed percentage deviation of one regression slope from the full-HF reference, using a different starting-day offset. The zero line denotes the full-HF reference. Reference ODRs were −0.0170, −0.0187 and − 0.0319 mg L⁻^1^ d⁻^1^ for 2022, 2023 and 2024, respectively

Across 2022–2024, the within-year range of ODR deviations increased as the sampling interval increased from 7 to 30 days (Fig. 2; Table S3). The mean range width was 8.0 percentage points at 7 days, 20.5 at 14 days, 47.5 at 30 days and 48.6 at 60 days. Individual schedules yielded both faster and slower depletion estimates relative to the full-HF reference. The increase was not strictly monotonic in every year, and the 60-day result was based on fewer estimable schedules.

The number of viable subsampling schedules varied considerably across years due to interannual differences in the duration of summer stratification. In 2023, an extended stratification period permitted ODR estimation for nearly all simulated schedules (58 of 60). In contrast, shorter stratification in 2022 and 2024 limited viable estimations to 22 and 28 schedules, respectively. Because all bimonthly and several monthly schedules relied on only two sampling dates, traditional regression goodness-of-fit metrics could not be computed. Deviation from the full-HF ODR was therefore used as the primary measure of sensitivity.

Using the actual LF sampling dates, the 2022 date-matched HF reconstruction did not reproduce the weaker LF ODR. ODRs were − 0.0170 mg L⁻^1^ d⁻^1^ for the full-HF series, − 0.0178 mg L⁻^1^ d⁻^1^ for the HF series retained on LF dates and − 0.0066 mg L⁻^1^ d⁻^1^ for the LF reconstruction (Fig. S3). In 2024, the date-matched HF and LF ODRs were both − 0.025 mg L⁻^1^ d⁻^1^, compared with − 0.032 mg L⁻^1^ d⁻^1^ for the full-HF reference.

### Depth-specific low-oxygen duration

**Fig. 3:**
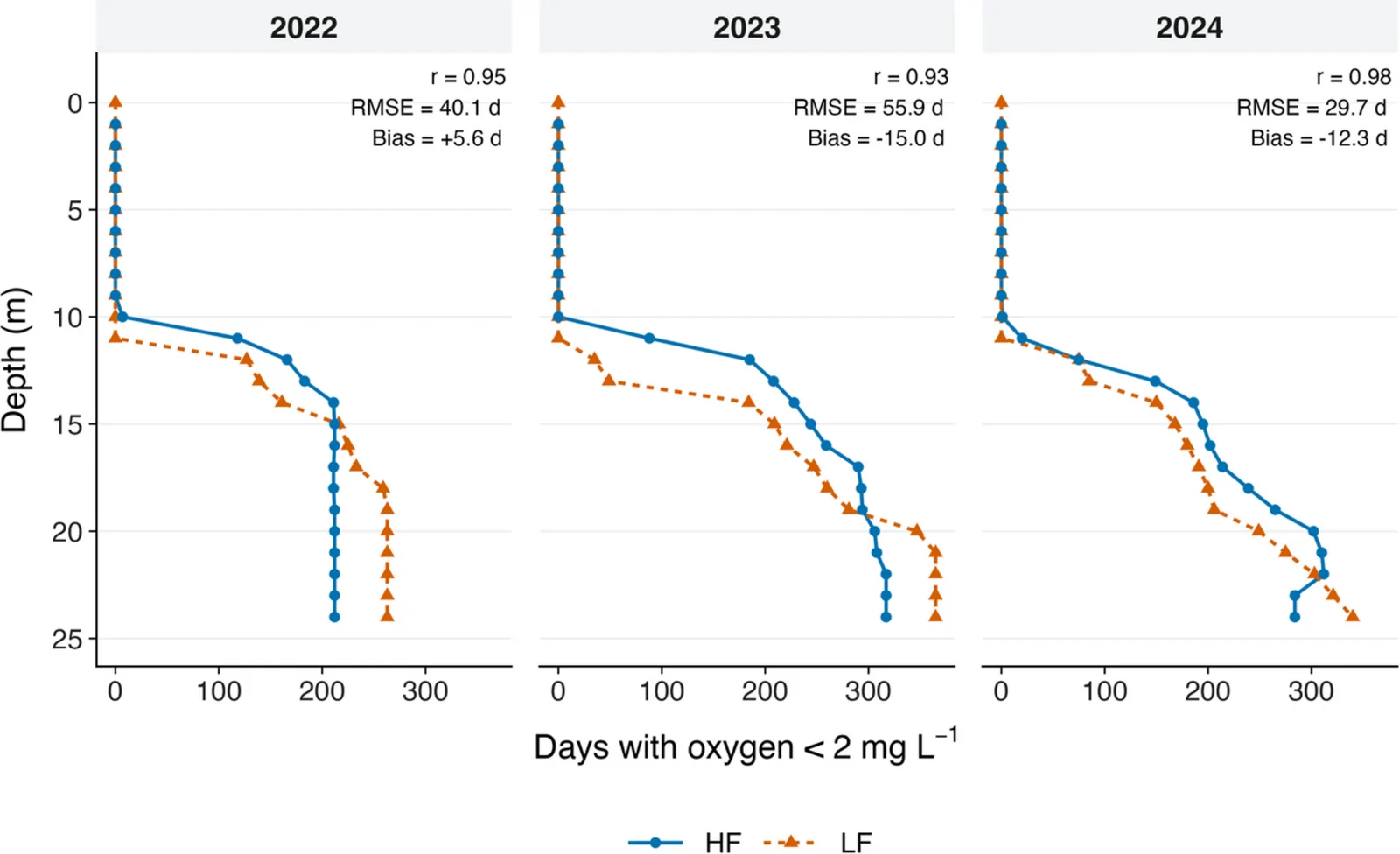
Depth-specific counts of days with oxygen < 2 mg L⁻^1^ in 2022–2024, derived from HF and LF daily records. Solid lines and circles denote HF observations; dashed lines and triangles denote LF estimates. Pearson’s *r* is calculated across paired depths. RMSE and bias are given in days, with bias defined as LF–HF. Counts use the days available in each dataset and are not gap-controlled full-year estimates

Depth profiles of the number of days with oxygen < 2 mg L⁻^1^ differed between HF and LF datasets (Fig. 3). RMSE across depths was highest in 2023, followed by 2022 and 2024, whereas the vertical pattern was strongly correlated between datasets.

The number of low-oxygen days generally increased with depth. No daily mean < 2 mg L⁻^1^ occurred at depths shallower than 10 m in either dataset. The shallowest affected HF depths were 10 m in 2022, 11 m in 2023, and 10 m in 2024; the shallowest affected LF depth was 12 m in all three years. At the deepest depths, counts approached the number of available days. The HF record contained 212, 317 and 324 available days in 2022, 2023 and 2024, respectively, whereas the LF record contained 263, 365 and 340 days. These counts imply different observation coverage and therefore cannot be interpreted as directly comparable annual durations. Depth-wise Pearson correlations between HF and LF low-oxygen counts were 0.947, 0.933 and 0.977 in 2022, 2023 and 2024, respectively, using 24 paired depths per year. RMSE values were 40.1, 55.9 and 29.7 days, and mean biases (LF–HF) were + 5.6, − 15.0 and − 12.3 days. In 2022, LF counts exceeded HF counts by 5.6 days on average across depths; mean differences were negative in 2023 and 2024.

### Comparison at original LF sampling dates

The depth-specific comparison included 229 paired date–depth observations from 29 LF sampling dates (Fig. S4). The largest persistent discrepancy occurred at 9 m, where mean HF − LF was − 2.49 mg L⁻^1^, RMSE was 3.10 mg L⁻^1^ and the maximum absolute difference was 6.61 mg L⁻^1^ (5 November 2024). At 12 m, mean bias was − 1.42 mg L⁻^1^ and RMSE was 2.02 mg L⁻^1^. RMSE values were 0.79 mg L⁻^1^ at 3 m, 0.77 mg L⁻^1^ at 6 m, 0.79 mg L⁻^1^ at 15 m, 1.31 mg L⁻^1^ at 18 m, 0.54 mg L⁻^1^ at 21 m and 0.23 mg L⁻^1^ at 24 m. Thus, disagreement was strongly depth-dependent, with the largest RMSE at 9 m.

## Discussion

Monthly profiles interpolated to daily resolution reproduced the broad seasonal trajectories of volume-weighted oxygen in all three operational layers (Fig. 1). This indicates that low-frequency profiles can retain information on seasonal oxygen dynamics, when the underlying changes are gradual relative to the sampling interval. The result is relevant to long-term lake monitoring programmes like in Lake Piburg because historical oxygen records are often based on discrete profiles, whereas continuous sensors are a comparatively recent addition to monitoring planning. High-frequency measurements nevertheless resolve diel variability and short-lived physical or biogeochemical events that cannot be reconstructed from monthly observations alone (Marcé et al., 2016; Meinson et al., 2016; Rozemeijer et al., 2025). Thus, the value of increased temporal resolution depends on the metric being estimated rather than on dissolved oxygen concentration alone.

The greater sensitivity of ODR to sampling interval is consistent with the different information requirements of concentration- and rate-based metrics (Fig. 2; Table S3). A sustained seasonal trajectory can be represented adequately by relatively sparse observations, whereas a rate estimate depends on the temporal placement of the observations used to define the trajectory. Moreover, hypolimnetic oxygen depletion is not necessarily linear throughout stratification. For example, approximately linear declines can characterize the higher-oxygen part of the depletion trajectory, whereas the decline becomes increasingly non-linear as oxygen concentrations approach zero (Yuan & Jones, 2019). The observed depletion rate also integrates water-column and sediment oxygen consumption and can be modified by mixing, renewal and other physical processes (Matzinger et al., 2010, Müller et al., 2012). Consequently, an ODR derived from a small number of widely spaced observations should be regarded as a sampling-dependent description of the oxygen trajectory rather than as a direct estimate of a single biogeochemical process. Long-term observations from other lakes likewise show that ODR varies with depth, trophic state, oxygen concentration and other environmental characteristics (Rhodes et al., 2017).

The present resampling experiment of the HF dataset showed that sampling timing becomes increasingly important as the interval between observations increases, whereas the effect was smaller at shorter intervals (Fig. 2; Table S3). The analysis does not identify a universal optimal sampling interval because it was based on predefined depletion windows in a single lake. Nevertheless, it shows that monitoring frequency should be selected according to the intended endpoint. Similar considerations have been emphasized in lake-monitoring studies that distinguish the frequency required to detect long-term change from that needed to resolve short-term variability (Patil et al., 2024), and in recent guidance advocating objective-specific high-frequency monitoring, explicit treatment of sensor uncertainty and systematic handling of data gaps (Rozemeijer et al., 2025). For Lake Piburg, monthly profiling appears sufficient for seasonal context, whereas more frequent observations would reduce the dependence of ODR estimates on sampling timing.

The resampling analysis also assumes that the depletion period is known. Fitting windows were selected from the HF record and then held fixed, so the analysis does not test whether low-frequency observations alone would identify the same onset and termination of depletion. This is an important practical distinction because a monitoring programme based only on discrete profiles must identify both the temporal trajectory and the period over which a rate is estimated. The supplementary comparison using the actual LF sampling dates illustrates the additional influence of reconstruction and measurement method (Fig. S3). Because that comparison combines temporal sampling with differences between in situ sensing and discrete Winkler measurements, it cannot assign the remaining discrepancy to a single cause. The HF subsampling experiment is therefore the more direct test of temporal sampling effects.

The vertical pattern of low-oxygen exposure was more robust than the absolute duration of exposure (Fig. 3). This is consistent with the different temporal information contained in threshold counts and concentration trajectories, namely, a persistent oxygen deficit extending over much of the stratification period is less sensitive to the exact sampling dates than a rate calculated from the slope between individual observations. However, the HF and LF records had different temporal coverage, limiting the interpretation of the counts as annual exposure durations. The strongest practical implication is therefore that sparse profiles can identify the depth range affected by prolonged low oxygen, but absolute duration requires a common observation window and explicit treatment of missing days.

The discrepancies between HF and LF concentrations further show that temporal agreement does not imply agreement at individual depths (Fig. S4). The largest differences occurred near 9 and 12 m, close to the operational boundary between the metalimnion and hypolimnion (Table S2). This pattern is consistent with the greater physical and biogeochemical heterogeneity of the metalimnion, where strong gradients and interacting processes can produce pronounced spatial variability (Giling et al., 2017). Interpolation can also perform differently depending on profile structure and comparisons of lake profile interpolation methods have shown that errors increase in heterogeneous profiles and can be pronounced near steep gradients (North & Livingstone, 2013). In the present study, differences in sampling depth, the finite vertical integration of the Schindler–Patalas sampler, movement of the transition zone, spatial separation between sensors and the sampler and analytical uncertainty may all contribute to the observed HF–LF mismatch. However, the available data do not allow these sources to be separated quantitatively.

Taken together, the results support an objective-specific approach to oxygen monitoring. Discrete monthly profiles can provide useful information on seasonal and vertical oxygen structure and preserve comparability with long historical records. Higher-frequency observations become increasingly valuable when the objective is to estimate rates, identify the onset or termination of depletion or resolve short-lived reoxygenation events. This interpretation is consistent with the broader high-frequency monitoring literature, which emphasizes that increased temporal resolution is most valuable when the process of interest varies on timescales shorter than the routine sampling interval (Marcé et al., 2016; Rozemeijer et al., 2025; Staehr et al., 2010). The present study provides evidence for metric-specific sensitivity to temporal sampling rather than a prescription for a particular sampling frequency.

The generality of our findings remains uncertain because the analysis was conducted in one small, dimictic, seasonally stratified lake. Oxygen dynamics vary among lakes with differences in depth, trophic state, mixing regime and stratification phenology. Multilake observations and modelling have shown that changes in stratification duration can substantially affect hypolimnetic oxygen conditions, demonstrating that the temporal structure of oxygen depletion is itself sensitive to lake-specific physical and climatic conditions (Ladwig et al., 2021; Robbins et al., 2025; Schwefel et al., 2025). The transferability of the sampling-frequency effects observed here should therefore be tested across lakes differing in morphometry, mixing regime, trophic state and oxygen dynamics.

## Conclusions

Monthly oxygen profiles reproduced the broad seasonal dynamics and the vertical distribution of prolonged low-oxygen conditions in Lake Piburg, indicating that low-frequency monitoring can remain informative for seasonal assessments and long-term records. In contrast, hypolimnetic oxygen depletion rates were substantially more sensitive to sampling frequency and sampling timing, particularly when observations were widely spaced within the depletion period. Thus, the temporal resolution required for oxygen monitoring depends on the metric being estimated rather than on dissolved oxygen concentration alone.

The comparison between HF and LF records also showed that good agreement at the scale of volume-weighted layers can coexist with substantial discrepancies at individual depths, particularly near strong vertical gradients. Moreover, unequal temporal coverage limited the interpretation of threshold-based oxygen duration estimates. These findings support the continued value of monthly profiling for broad seasonal and long-term assessments, while more frequent observations are warranted when the objectives include rate estimation, identification of depletion or reoxygenation dynamics or characterization of short-lived events. Because the study was conducted in a single small, dimictic lake, the observed sampling-frequency effects should be evaluated across lakes with different morphometry, mixing regimes and oxygen dynamics before being generalized.

## Supplementary Information

Below is the link to the electronic supplementary material.

ESM 1 PDF (647 KB)

## Data Availability

The raw uninterpolated datasets are available from Zenodo (DOI: https://zenodo.org/records/20559145).

## Declarations

All authors have read, understood and have complied as applicable with the statement on “Ethical responsibilities of Authors” as found in the Instructions for Authors.
