# Supplementary material for: Robustness of oxygen metrics to sampling frequency in a stratified lake
Source: Environ Monit Assess. 2026 Oct 21;198(11):1239. doi: 10.1007/s10661-026-16081-8 (PMC13687420; doi:10.1007/s10661-026-16081-8)
Supplement: Supplementary file 1 [file 10661_2026_16081_MOESM1_ESM.pdf]

Supplementary Information for

Robustness of oxygen metrics to sampling frequency in a stratified lake

Eric Weniger & Ruben Sommaruga

Department of Ecology, Universität Innsbruck, Technikerstr. 25, 6020 Innsbruck, Austria

Corresponding author: ruben.sommaruga@uibk.ac.at

Content

Supplementary Methods:

Temporal oxygen patterns and ODR comparison; Tables S1–S2

Supplementary Results:

Table S3; Figures S1–S4

Supplementary Methods

Temporal oxygen patterns

To describe temporal structure in the HF record, daily volume-weighted oxygen concentrations were modelled as a function of time and lake layer (epilimnion, metalimnion, and hypolimnion) using a generalized additive model (GAM). Time was represented by separate layer-specific smooths using factor-by smooths, and lake layer was included as a parametric effect. Models were fitted in R with the mgcv package, assuming Gaussian errors with an identity link; smoothing parameters were estimated by restricted maximum likelihood (REML). An initial model was fitted with gam(), and the final model used bam() with rho and AR.start to account for first-order temporal dependence. A separate autoregressive series was started after gaps >1 day. We used rho = 0.85 and k = 20 for each layer-specific smooth. The GAM was used only to describe broad seasonal and layer-specific dynamics (Fig. S2); it did not replace observed HF values in any HF–LF comparison or ODR calculation.

ODR comparison

To compare ODR estimates using actual LF sampling dates (Fig. S3), we applied the HF-defined depletion windows to three series: the full HF daily record; HF observations retained only on LF sampling dates and reconstructed to daily resolution; and the actual LF record reconstructed to daily resolution. The first two series isolate the effects of retaining LF sampling dates and temporal reconstruction. Comparison with the LF record also includes differences in measurement method and therefore does not isolate sampling-frequency effects. For the date-matched HF reconstruction, whole-day HF means were retained only on dates when an LF profile was collected. At each 1 m depth, the retained HF observations were interpolated to daily resolution using the same cubic-spline approach applied to LF data; volume-weighted hypolimnetic means were then calculated for depths ≥10 m. No extrapolation beyond the available observations was used.

Linear regressions were fitted only on dates with finite values in all three series within the HF-defined windows. This shared-date requirement restricted the fitting periods to 25 April–15 July 2022, 12 February–9 June 2023, and 11 April–23 July 2024, with 82, 118, and 88 shared daily observations, respectively. The same fitting periods and full-HF reference slopes were used for the HF subsampling analysis. For each series and year, ODR was estimated as the ordinary least-squares slope of hypolimnetic oxygen concentration against elapsed time in days. Model fit was described by R^2 and Pearson's r; no significance tests were performed because the daily values are temporally dependent and partly reconstructed.

Table S1 LF sampling dates for Lake Piburg, 2022–2024.

LF sampling date
2022-01-13
2022-02-03
2022-03-07
2022-04-04
2022-04-25
2022-05-12
2022-06-08
2022-07-06
2022-08-03
2022-09-02
2022-10-06
2022-11-15
2022-12-09
2023-01-30
2023-02-15
2023-03-06
2023-04-12
2023-05-04
2023-06-01
2023-07-03
2023-08-01
2023-09-06
2023-10-02
2023-11-08
2023-12-05
2024-01-17
2024-03-04
2024-04-02
2024-05-06
2024-06-05
2024-07-02
2024-08-05
2024-09-09
2024-10-07
2024-11-05
2024-12-04

Table S2 Temperature-transition endpoint depths from daily temperature profiles during 2022–2024. Values are medians (25th–75th percentiles). HF, high frequency; LF, low frequency.

Temperature dataset	Qualifying days	Upper endpoint (m)	Lower endpoint (m)
HF daily averages	595	4 (2–4)	9 (6–9)
LF daily interpolated profiles	533	4 (4–7)	9 (9–9)

Supplementary results

Table S3 Signed deviation ranges from the full-HF ODR by sampling interval (2022–2024).

Sampling interval	Year	Signed deviation range (%)	Mean within-year range width (percentage points)
7-day	2022	−2.9 to +2.9	8.0
	2023	−2.9 to +2.8	
	2024	−5.7 to +6.7	
14-day	2022	−12.9 to +14.6	20.5
	2023	−6.6 to +3.8	
	2024	−14.5 to +9.2	
30-day	2022	−15.9 to +48.2	47.5
	2023	−19.7 to +10.7	
	2024	−34.1 to +13.9	
60-day	2022	−15.9 to +12.8	48.5
	2023	−26.5 to +32.3	
	2024	−28.2 to +29.9	

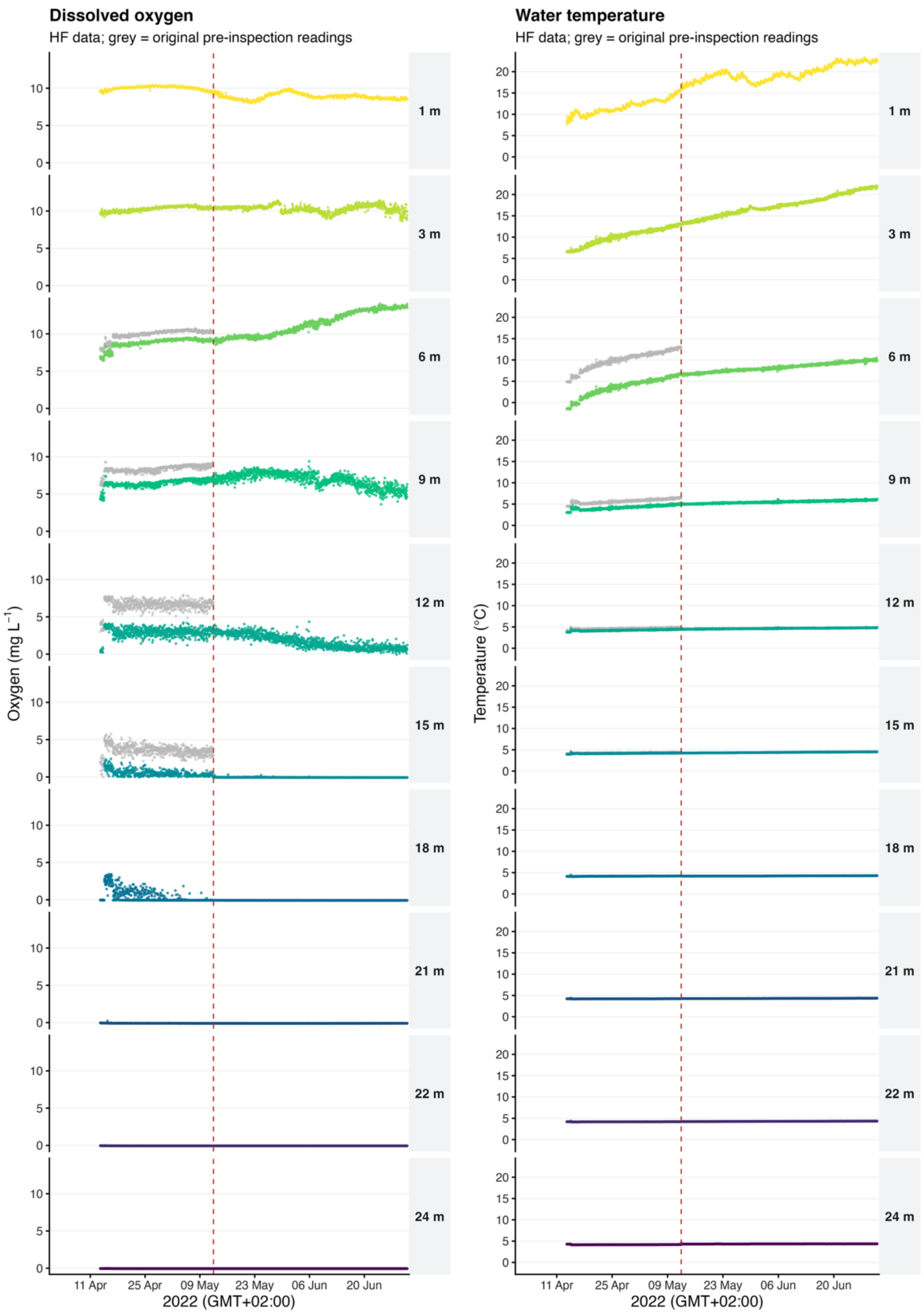

Fig. S1 Sensor offset correction in April–June 2022. Points show hourly oxygen and temperature observations; original pre-inspection readings at 6, 9, 12, and 15 m are shown separately. The dashed line marks the inspection on 12 May at 11:00 CEST (09:00 UTC). Constant offsets applied before inspection align the preceding and following 24-h medians. Post-inspection observations and other depths were unchanged. Oxygen values that became negative after correction were treated as missing before interpolation and averaging. The plotted observations are uninterpolated.

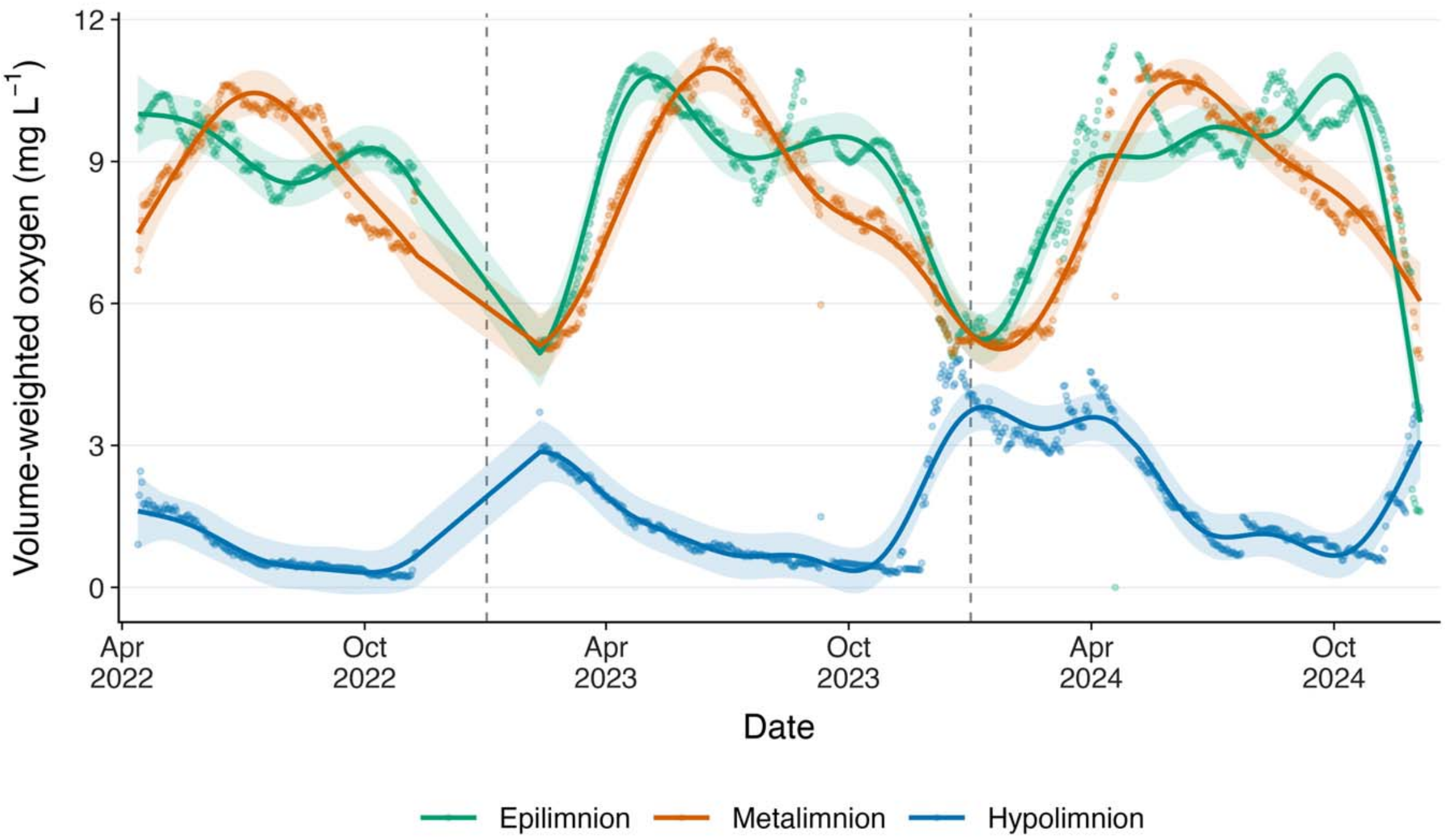

Fig. S2 HF time series. Points show daily volume-weighted oxygen concentrations. Lines show GAM predictions and shaded bands show model confidence intervals. Separate lines represent the epilimnion, metalimnion, and hypolimnion. Vertical dashed lines mark the start of each year.

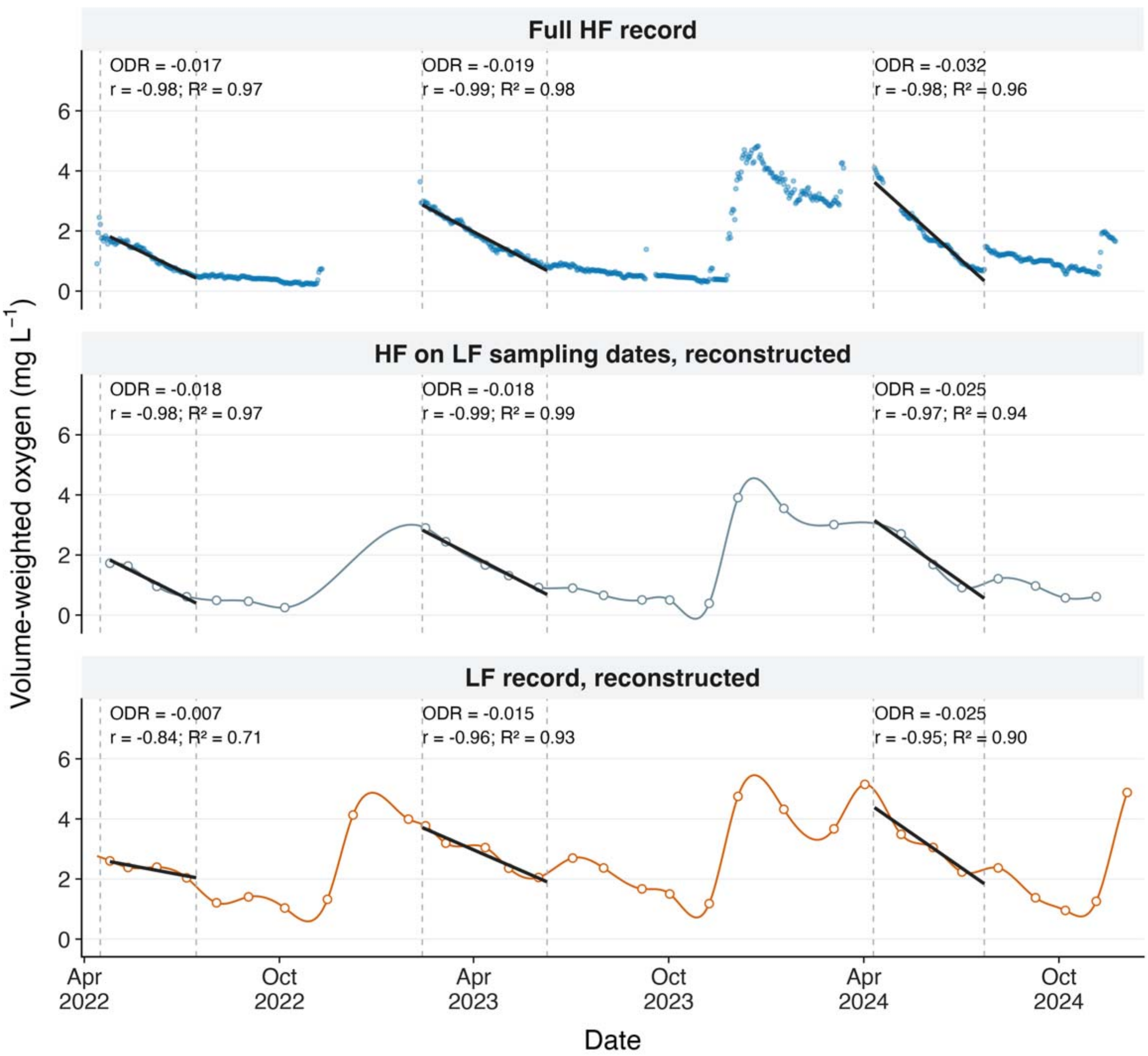

Fig. S3 Hypolimnetic oxygen depletion rates (ODR) calculated from the full HF record, HF observations retained on actual LF sampling dates and reconstructed to daily resolution, and the actual LF record reconstructed to daily resolution. Open points show retained sampling dates. Vertical dashed lines delimit the HF-defined depletion windows; black lines are linear regressions fitted to dates available in all three series. ODR is given in mg L^{-1} d^{-1}. Pearson's r is the correlation between oxygen concentration and elapsed time. Fits are descriptive; uncertainty intervals are omitted because temporal dependence and interpolation are not accounted for.

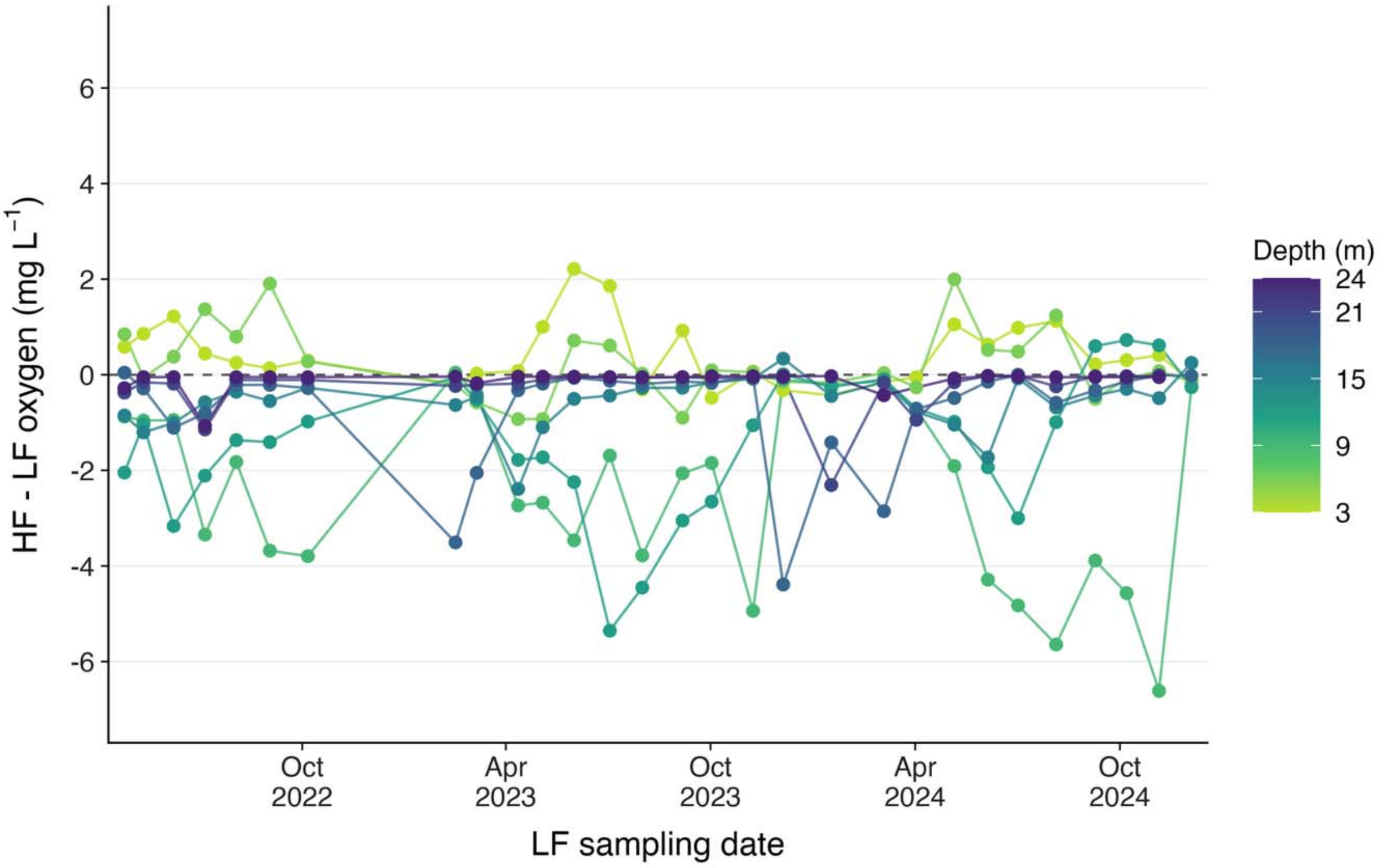

Fig. S4 Differences in oxygen concentration (HF − LF) at original LF sampling dates and identical nominal depths. HF values are means from 08:00 to 17:00 local time (Europe/Vienna); LF values are original observations without vertical or temporal interpolation. The dashed horizontal line indicates zero difference; negative values indicate higher LF concentrations. Lines connect observations at the same depth.
